# Detection of a Conspecific Mycovirus in Two Closely Related Native and Introduced Fungal Hosts and Evidence for Interspecific Virus Transmission

**DOI:** 10.3390/v10110628

**Published:** 2018-11-13

**Authors:** Corine N. Schoebel, Simone Prospero, Andrin Gross, Daniel Rigling

**Affiliations:** Swiss Federal Institute for Forest, Snow and Landscape Research, WSL, Zuercherstrasse 111, 8903 Birmensdorf, Switzerland; Simone.Prospero@wsl.ch (S.P.); andrin.gross@wsl.ch (A.G.); Daniel.rigling@wsl.ch (D.R.)

**Keywords:** *Chalara fraxinea*, *Hymenoscyphus pseudoalbidus*, ash dieback, Narnaviridae, evolution, invasive species, horizontal virus transmission

## Abstract

*Hymenoscyphus albidus* is a native fungus in Europe where it behaves as a harmless decomposer of leaves of common ash. Its close relative *Hymenoscyphus fraxineus* was introduced into Europe from Asia and currently threatens ash (*Fraxinus* sp.) stands all across the continent causing ash dieback. *H. fraxineus* isolates from Europe were previously shown to harbor a mycovirus named *Hymenoscyphus fraxineus Mitovirus 1* (HfMV1). In the present study, we describe a conspecific mycovirus that we detected in *H. albidus*. HfMV1 was consistently identified in *H. albidus* isolates (mean prevalence: 49.3%) which were collected in the sampling areas before the arrival of ash dieback. HfMV1 strains in both fungal hosts contain a single ORF of identical length (717 AA) for which a mean pairwise identity of 94.5% was revealed. The occurrence of a conspecific mitovirus in *H. albidus* and *H. fraxineus* is most likely the result of parallel virus evolution in the two fungal hosts. HfMV1 sequences from *H. albidus* showed a higher nucleotide diversity and a higher number of mutations compared to those from *H. fraxineus*, probably due to a bottleneck caused by the introduction of *H. fraxineus* in Europe. Our data also points to multiple interspecific virus transfers from *H. albidus* to *H. fraxineus*, which could have contributed to the intraspecific virus diversity found in *H. fraxineus*.

## 1. Introduction

In the past years, species invasions have become an important topic in the scientific literature due to increasing awareness of the consequences of intensified international trade of plant material [1,2,3]. In the case of introduced (exotic) plant pathogens, we frequently observe invasions by species that have a harmless close relative in the invaded area and which are often more virulent due to the lack of co-evolution with the novel host. This can have catastrophic impacts on forest ecosystems (e.g., [4,5]). So far, most studies have focused on the invasive species itself, while little attention has been paid to possible interactions with closely related native species that may occupy similar ecological niches and share evolutionary histories. The duration of this interaction is often limited because the invasive species outcompetes its native sister species (e.g., *Cryphonectria parasitica* vs. *C. radicalis* in Europe; [6]). Nevertheless, during this short timeframe, gene flow between native and introduced species may occur, resulting in varying levels of gene introgression between the two interacting taxa. The occurrence of hybridization between allopatric species following attained sympatry because of anthropogenic introduction has been reported for example in the basidiomycete genus *Heterobasidion* [7] and in the oomycete genus *Phytophthora* [8].

In the present study we focus on the interaction of *Hymenoscyphus fraxineus* and *H. albidus*, which belong to the family Helotiaceae and are closely related [9]. The first species is native to Asia and in Europe is an invasive pathogen causing ash dieback (ADB), mainly on common (*Fraxinus excelsior*) and narrow-leaved ash (*Fraxinus angustifolia*) [10]. The latter species is a native endophyte of common ash in Europe (e.g., [11]). Due to its severe ecological consequences, there has lately been a great interest in the biology, ecology, and population genetics of *H. fraxineus* (e.g., [12,13,14,15,16]). However, little is known about *H. albidus* [17,18], as well as its interaction with *H. fraxineus*. Field reports indicate that since the onset of the ADB epidemic in Europe, the frequency of detection of *H. albidus* has decreased strongly, with the species being at risk of local extinction [19,20]. Both species show a similar life cycle and, more importantly, occupy the same sporulation niche, i.e., rachis and petioles of last year’s ash leaves in the litter [17,18,20]. The introduced *H. fraxineus* is most likely outcompeting its native relative because of its superior ability to cause leaf infections and its massive production of airborne ascospores [21]. After the first detection of the disease in the 1990s in Eastern Europe [22], *H. fraxineus* is now abundant in most ash stands all across mainland Europe and the UK [15,23,24].

Fungal viruses (mycoviruses) are widespread in all major groups of fungi [25] and a mycovirus, *Hymenoscyphus fraxineus Mitovirus 1* (HfMV1) was recently also discovered in *H. fraxineus* [26]. HfMV1 is a putative member of the genus Mitovirus (family *Narnaviridae*) that are only present in fungi. Mitoviruses are commonly found in fungi and represent the simplest mycoviruses with an unencapsidated single strand RNA genome of approximately 2.5 kb. The genome contains one single open reading frame (ORF), encoding the RNA-dependent RNA polymerase (RdRp). As many other RNA viruses, mitoviruses are mutating at high rates [27]. Mitoviruses are located and translated in the mitochondria [28,29]. It has previously been shown that some mycoviruses may be transferred between related fungal species e.g., in the genera *Cryphonectria*, *Heterobasidion*, *Sclerotinia*, as well as between fungal taxa e.g., *Sclerotinia homoeocarpa* and *Ophiostoma novo-ulmi* [30,31,32,33]. In many of these studies, evidence for interspecific virus transmission was based on the occurrence of conspecific viruses in different fungal host species.

In the present study, we tested the occurrence of a conspecific mitovirus in *H. albidus* and *H. fraxineus* using the RdRP gene as a marker for population genetic analyses. Specifically, we (i) determined the presence and prevalence of HfMV1 sequences in *H. albidus* isolates, which were collected in Switzerland and France before the arrival and spread of the invasive *H. fraxineus*; (ii) determined the phylogenetic relationship of the viral sequences detected in *H. albidus* and *H. fraxineus*; and (iii) compared the genetic diversity of the viruses in *H. albidus* and *H. fraxineus* populations from the same geographic regions, to determine the possible origin of the conspecific virus in the two closely related host species.

## 2. Materials and Methods

### 2.1. Isolates Used in the Study

Overall, 67 isolates of *H. albidus* from France and Switzerland and 221 HfMV1-positive isolates of *H. fraxineus* from Europe (mainly Switzerland) were analyzed (Table 1). All *H. albidus* isolates were recovered from colonized petioles of common ash. The *H. fraxineus* isolates were also obtained from common ash, either from petioles or bark lesions (for details see [14,26]). All *H. albidus* isolates were identified by ITS sequencing using ITS1 and ITS4 primers [34]. The *H. fraxineus* isolates were identified either by ITS sequencing or microsatellite genotyping [12]

### 2.2. Fungal Cultivation and RNA Extraction

For each sample, a small piece of growing pure culture was transferred to a new cellophane covered malt agar plate [13]. These were incubated for 5 weeks in the dark at room temperature. Thereafter, the mycelia were harvested, lyophilized and then milled in a MixerMill (MM33, Retsch GmbH, Haan, NRW, Germany). For each sample, approximately 20 mg of the ground mycelium were used for RNA extraction, using the PureLink Pro 96 total RNA Purification Kit (Invitrogen, Carlsbad, CA, USA), following the manufacturer’s instructions.

### 2.3. Screening for HfMV1 and Viral Sequencing

All *H. albidus* and *H. fraxineus* isolates were screened for the presence of HfMV1 using a specific PCR assay [26]. Complementary DNA (cDNA) was produced with the Maxima First Strand cDNA Synthesis Kit (Life Technologies, Carlsbad, CA, USA) according to the manufacturer’s protocol. For sequencing, PCR products were purified using Illustra ExoProStar (GE Healthcare Life Sciences, Pittsburgh, PA, USA) according to the instructions. Sanger sequencing of the PCR products was done on both strands using the same primers as for PCR. In addition, for 40 isolates (8 for *H. albidus*, 32 for *H. fraxineus*) the full length viral RdRP gene (2151 bp) was sequenced in both directions as described in [26]. To account for mutations at primers sites in some of the isolates, additional primers had to be designed to obtain the complete ORF (see Table S2).

### 2.4. Phylogenetic Analyses

The maximum likelihood (ML) phylogenetic analysis was conducted for the nucleotide alignment. The alignment quality was first double-checked using the MACSE aligner [36]. A total of 40 separate runs each with 10,000 replicates were conducted using the ‘rapid bootstrap analysis and tree search’ algorithm, resulting in a best ML tree with branch lengths and bootstrap support values. Two separate analyses were performed, (i) for the full length RdRP gene (2151 bp, N = 40) and (ii) for the partial RdRP gene (495 bp, N = 255). For the partial RdRP gene, three distinct data partitions with joint branch length optimization in RAxML v8.2.4 [37] implementing the GTRGAMMA model of nucleotide substitution were used.

The Bayesian analysis was conducted for each of the two datasets separately, using MRBAYES v3.2.6 [38]. Three independent runs, each consisting of 100 million generations with a burn-in of 80 million and a sampling frequency of 1000 were performed.

The resulting data was visualized using the software TREEGRAPH v2 [39] and FIGTREE v1.4.2 (http://tree.bio.edac.uk/software/figtree/).

### 2.5. Genetic Diversity, Differentiation and Evolution

DNASP v5 [40] was used to determine the nucleotide diversity estimated by the average number of differences per site between two sequences (π), the number of haplotypes observed (h), the haplotype diversity (Hd), the number of segregating sites (S), the number of total mutations (η), and the average number of differences (K) per country/population were calculated for the two datasets ‘partial ORF’ and ‘full ORF’. Pairwise identity percentages for the nucleotide and amino acid sequences were calculated using Multiple Sequence Comparison by Log- Expectation (MUSCLE; http://www.ebi.ac.uk/Tools/msa/muscle).

## 3. Results

In the present study, we describe the first fungal virus detected in *H. albidus*. HfMV1, which was previously described for *H. fraxineus*, could now also be detected in its close relative, *H. albidus*. Although vast areas of Switzerland were sampled, from 2013 onward *H. albidus* could not be isolated any longer.

### 3.1. Prevalence of HfMV1 in H. albidus

**Figure 1:**
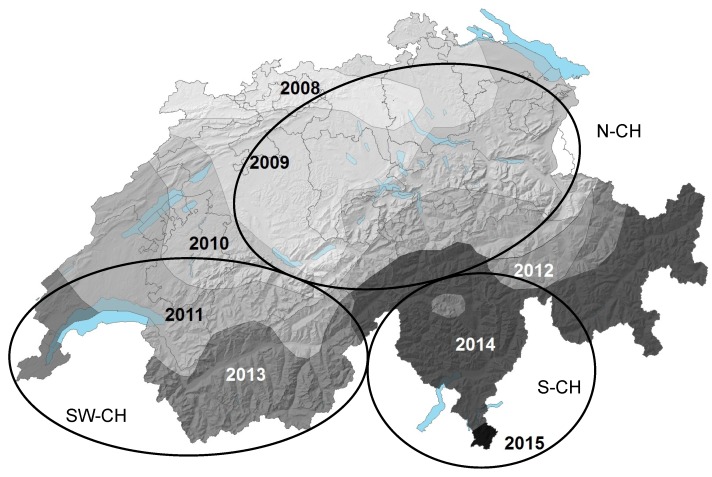
Geographic origin of the Swiss *Hymenoscyphus albidus* and *H. fraxineus* samples analyzed in this study. Grey tones display the year of first report of ash dieback. Light grey lines display Swiss canton borders. Abbreviations: S-CH: Southern Switzerland, SW-CH: South-Western Switzerland, N-CH: Northern Switzerland. Modified after [41].

All *H. albidus* isolates analysed originated from Swiss and French regions where *H. fraxineus* had not been officially reported at the time of sampling (Figure 1). By using a HfMV1-specific PCR assay, we detected HfMV1-sequences in 33 out of 67 *H. albidus* isolates (49.3%). More precisely, 28 out of 53 Swiss isolates (52.8%) and 5 out of 14 French isolates (35.7%) harbored HfMV1.

### 3.2. Pairwise Sequence Identity

All virus sequences obtained from *H. albidus* and *H. fraxineus* showed high similarities and were of identical length for both the full and partially sequenced RdRP gene. Average pairwise sequence identity (PWI) was 95% (range between 93% to 97%), when comparing the full ORF amino acid sequences (717 AA) of HfMV1 originating from the two fungal host species. For the nucleotide sequences (NT) the average PWI was 91% (90% to 93%). When considering only HfMV1 sequences from *H. albidus*, the PWI value was on average 97% (92% for NT), whereas for HfMV1 sequences from *H. fraxineus*, the PWI value was 98% (96% for NT). According to the International Committee on Taxonomy of Viruses, a PWI < 40 indicates different species, whereas PWI > 90 is indicative that the compared sequences belong to the same virus species [42]. Therefore, we can conclude that all viral sequences analyzed in this study belong to the same species, which was previously described as HfMV1 [26].

### 3.3. Phylogenetic Relationships of HfMV1 across Two Different Host Species

**Figure 2:**
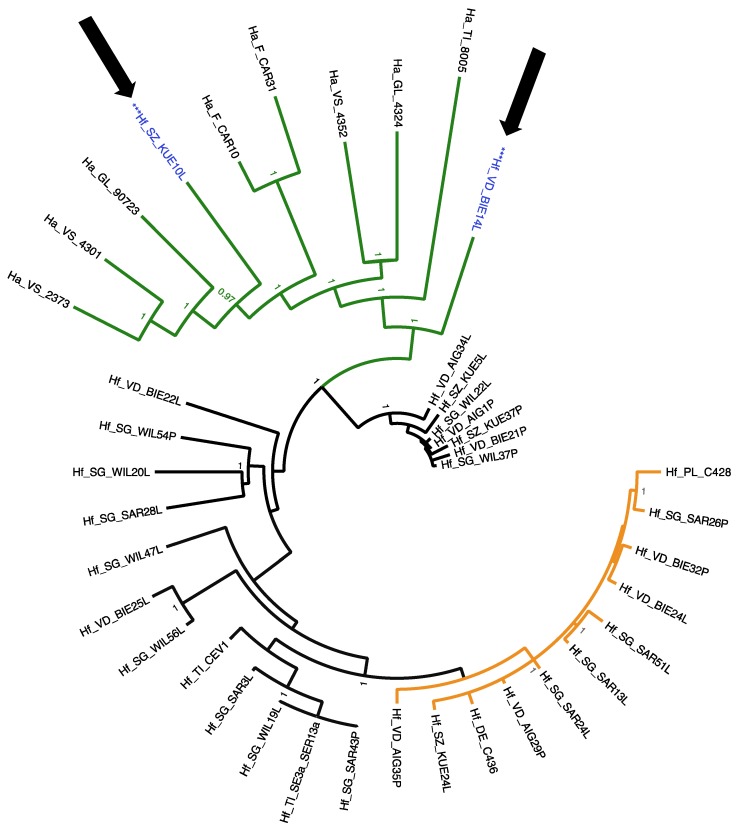
Phylogenetic tree resulting from the MrBayes analysis conducted with 40 (8 *H. albidus*, 32 *H. fraxineus*) full sequences (2151 bp) of the RdRP gene of the mitovirus HfMV1. Green color depicts HfMV1 *H. albidus* group isolates. Black color depicts HfMV1 group 2 isolates and orange color HfMV1 group 1 isolates. Blue color and *** marks *H. fraxineus* isolates within the *H. albidus* group. Hf characterizes viral sequences from *H. fraxineus*, Ha from *H. albidus* isolates. For abbreviation see Table 1. Posterior probabilities are shown at the respective branch if they were >0.95. Arrows indicate intermixing of *H. albidus* and *H. fraxineus* HfMV1 sequences.

For the full RdRP gene of HfMV1 (2151 bp), three well supported groups of sequences were detected (posterior probability, PP = 1; Figure 2). RAxML analysis evidenced very similar three groups, with >90% bootstrap support (Figure S1). In accordance with [15], these groups were called HfMV1 group 1, HfMV1 group 2 and HfMV1 *H. albidus* group. All viral sequences obtained from *H. albidus* belonged to a separate group, which consisted of long-branched sequences. HfMV1 group 2 also included long-branched sequences, all from Switzerland. HfMV1 group 1, on the other hand, was characterized by short branches with little differentiation and low support values. The sequences of the German and Polish isolates, which were initially used for HfMV1 primer design [26], came to reside in this group (Hf_DE_C436 and Hf_PL_C428 in Figure 2). The full RdRP sequences from *H. fraxineus* were allocated both to HfMV1 group 1 (11 isolates, light grey in Figure 2) and HfMV1 group 2 (19 isolates, black in Figure 2). All 8 full RdRP sequences obtained from *H. albidus* formed the *H. albidus* group. In addition, also two full RdRP sequences of *H. fraxineus* isolates obtained from necrotic lesions cluster here (arrows and blue font in Figure 2).

**Figure 3:**
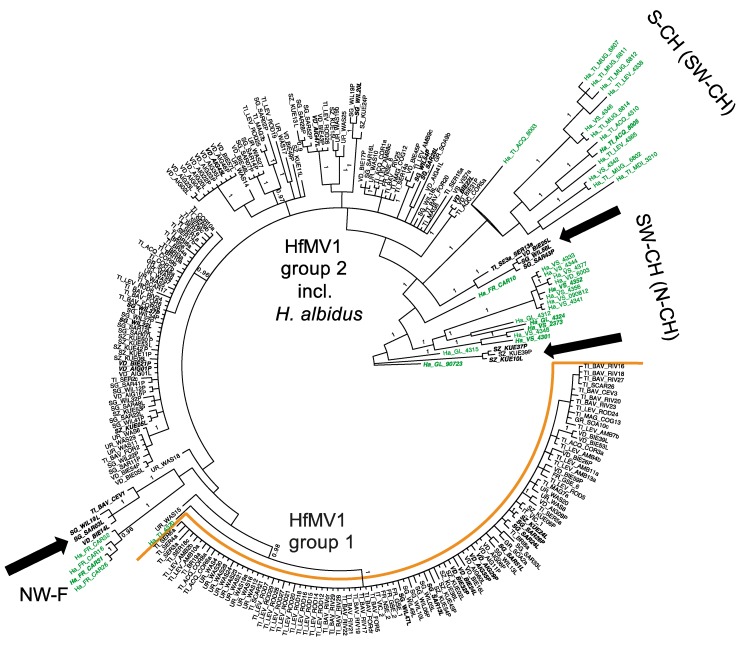
MrBayes analysis conducted with 255 sequences of the partial RdRP gene (495 bp) of the mitovirus HfMV1. Green color depicts *H. albidus* isolates. Posterior probabilities are shown at the respective branch if they were >0.95. Each sample is named with the sampling year, followed by the sampling locality (for abbreviation see Table 1) and sample name. Arrows indicate intermixing of *H. albidus* and *H. fraxineus* HfMV1 sequences. Samples marked in bold and italics are included in the full-length analysis.

For the partial RdRP gene of HfMV1 (495 bp), two major groups of sequences (PP = 1, bootstrap value 61%) were detected (Figure 3). In accordance with the full RdRP gene sequences and [14] these groups were again called HfMV1 group 1 and HfMV1 group 2. The *H. albidus* sequences formed three distinct clusters, which were all closer related to HfMV1 group 2 than to HfMV1 group 1 (Figure 3).

No structure could be detected in the overall HfMV1 population (i.e., combining *H. albidus* and *H. fraxineus*), neither geographically nor chronologically, using area or sampling year as priors. Nonetheless, within HfMV1 sequences of *H. albidus*, a certain geographic clustering was visible, with French (NW-F) isolates grouping together most distinctly (Figure 3). The 12 HfMV1 sequences from Southern Switzerland, together with two sequences from South-Western Switzerland (SW-CH) formed the second most distinct cluster (Figure 3 and Figure S2). Most sequences from SW-CH also grouped together in a separate cluster. Noteworthy, 12 *H. fraxineus* HfMV1 sequences grouped within the three *H. albidus* clusters (indicated by black arrows in Figure 3).

### 3.4. Population Genetic Parameters

#### 3.4.1. Full RdRP Gene

Given that all full-length RdRP sequences were different, the haplotype diversity (Hd) was one in both *H. fraxineus* and *H. albidus* (Table 2). The nucleotide diversity estimated by the average number of differences per site between two sequences (π) was 0.03 across all HfMV1 sequences from both host species for the full ORF (2151 bp; N = 40; Table 2). Among HfMV1 sequences from *H. albidus*, π was 0.077, and 0.021 for *H. fraxineus* sequences. The average number of nucleotide differences K, was 52 across all sequences from both species and ranged between 38 in *H. fraxineus* and 163 in *H. albidus*. The total number of mutations (η) was 380 for the entire population (*H. fraxineus* and *H. albidus* combined) and was lower in the Swiss *H. fraxineus* (238) than in the Swiss *H. albidus* population (403; Table 2).

#### 3.4.2. Partial RdRP Gene

For the partial RdRP sequences (495 bp; N = 255), π was on average 0.02 (0.08 for *H. albidus* and 0.007 for *H. fraxineus*; Table 3). Hd was on average 0.84 and ranged between 0.90 and 1.00 in *H. albidus* and between 0.7 and 0.99 in *H. fraxineus*. K, the average number of nucleotide differences, was 5 across all sequences from both species and ranged between 3 in *H. fraxineus* and 30 in *H. albidus*, all from Southern Switzerland (S-CH; Figure 1). The total number of mutations (η) was 89 for the entire population (*H. fraxineus* and *H. albidus* combined) and lower in Swiss *H. fraxineus* (58) than in Swiss *H. albidus* populations (122; Table 3).

## 4. Discussion

The main objective of this study was to determine the presence and prevalence of the mitovirus HfMV1 [26] in the leaf endophyte *H. albidus* and to use the viral RdRP gene for population genetic analyses. Furthermore, we aimed to compare the genetic diversity of HfMV1 in *H. albidus*, which is presumably native to Europe, to that in the closely related and introduced fungus *H. fraxineus*, the causal agent of ash dieback (ADB). Both fungi occupy the same ecological niche, colonizing and infecting ash leaves and forming fruiting bodies on leaf petioles in the litter. While *H. fraxineus* is very abundant, *H. albidus* is now difficult to find and possibly locally extinct [19,20]. Our study supports this observation, as from 2013 onwards we could only isolate *H. fraxineus* even in areas where *H. albidus* had previously been officially reported. It is generally assumed that this is the result of a displacement of *H. albidus* due to the competition for the sporulation niche with its relative *H. fraxineus*. As *H. fraxineus* acts as a primary leaf pathogen and produces massive airborne spore clouds (see also [21]), it is outcompeting the native *H. albidus*.

Genetic analyses confirmed that the mitovirus detected in *H. albidus* effectively corresponds to the mitovirus HfMV1 previously described in the invasive pathogen *H. fraxineus*. First, the viral RdRP gene has an identical ORF length in both fungi. Second, the full RdRP gene sequences obtained from *H. albidus* and *H. fraxineus* showed high pairwise identities (94.5% for the RdRP amino acid sequences, 91.1% for nucleotide sequences). As this finding indicates that HfMV1 is conspecific in *H. fraxineus* and *H. albidus*, we propose the same virus name, HfMV1, for both fungal species. This is the first description of a fungal virus in the leaf endophyte *H. albidus*.

The overall prevalence of HfMV1 in *H. albidus* is lower (49.3%) than previously described for *H. fraxineus* isolates from the European mainland and Great Britain (78.7% and 67%, respectively; [15,24]). This difference could be due to the fact that we analyzed far more *H. fraxineus* compared to *H. albidus* isolates. Furthermore, there could be a geographic difference, as the *H. albidus* isolates in this study were obtained mainly from Switzerland (and France), whereas the *H. fraxineus* isolates were collected throughout Europe (and Great Britain). The lower prevalence of HfMV1 in *H. albidus* could also be due to the PCR assay used for virus screening, which presumably did not detect all HfMV1 variants in this species. Nonetheless, the prevalence of HfMV1 in both *Hymenoscyphus* species is in accordance with the ranges observed for mitoviruses in other fungal species e.g., in *Gremmeniella* [43].

All *H. albidus* isolates analyzed in this study, including those infected by HfMV1, were collected in Switzerland and North-Western France prior to the arrival of ADB (Figure 1; [35]). Therefore, we can assume that HfMV1 must have been present in *H. albidus* before the introduction of *H. fraxineus* into Europe. This assumption is supported by the fact that *H. albidus* viruses form a distinct, well supported phylogenetic group when considering the full viral RdRP gene (Figure 2). The *H. albidus* viruses also exhibit a higher genetic diversity than the *H. fraxineus* viruses. Moreover, clustering by geographic region is evident for HfMV1 strains from *H. albidus*. Specifically, sequences from Switzerland are clearly separated from those from North-Western France. Within Switzerland, sequences from Southern Switzerland are quite distinct and only partially intermix with HfMV1 sequences from South-Western Switzerland. Such spatial genetic patterns are expected due to local differentiation of HfMV1 in native populations of *H. albidus*. In contrast, we did not detect any geographic structuring for HfMV1 sequences obtained from *H. fraxineus*, which is in accordance with previous studies for the fungus and the virus [12,13,14,15]. In summary, these findings suggest that HfMV1 is also native to Europe where it occurs as a genetically distinct group in *H. albidus*.

The presence of a conspecific mitovirus in *H. fraxineus* and *H. albidus* could be the result of a parallel virus evolution from a common ancestor. Mitoviruses are the most widespread fungal viruses and HfMV1only encodes one single gene. Hence, possibilities for mutations are limited and similar HfMV1 variants may form independently (in parallel) in different fungal hosts, particularly if the host species are closely related. Since mating between the two fungal species, which also possess different mating systems (homothallic in *H. albidus* vs. heterothallic in *H. fraxineus*), has never been observed [44], transfer of HfMV1 from *H. albidus* to *H. fraxineus* through interspecific hybridization does not seem to be a plausible hypothesis.

The detection of HfMV1 in *H. albidus* sheds new light on previous research, where only viral sequences obtained from *H. fraxineus* were investigated [14]. Based on the discovery of two HfMV1 groups in Europe, it was hypothesized that two divergent HfMV1 strains were introduced together with two *H. fraxineus* strains. The two HfMV1 groups in *H. fraxineus* were also detected in the present study for both the full and partial viral RdRP gene sequences. Although the *H. albidus* viruses are well separated from these two groups, they seem to be more closely related to HfMV1 group 2 than to group 1 (Figure 2 and Figure 3). Most interestingly, there are several HfMV1 sequences from *H. fraxineus*, which cluster together with *H. albidus* viruses, i.e., these viruses are closer related to those in *H. albidus* than to those in *H. fraxineus* (arrows in Figure 2 and Figure 3). This pattern is consistent with the hypothesis of an interspecies virus transmission from the native *H. albidus* to the invasive *H. fraxineus*. In particular, there are 2 full sequences of *H. fraxineus* isolated from necrotic lesions that cluster with the *H. albidus* group (Figure 2). Our results suggest at least three cross-species transmission events of HfMV1. Further interspecies virus transfers cannot be ruled out particularly within HfMV1 group 2, which could explain the higher genetic diversity observed in this group compared to group 1 [15]. In plant pathogenic fungi, cross-species transmission of mycoviruses has been previously described in different genera, e.g., *Cryphonectria hypovirus 1* (family Hypoviridae) in the genus *Cryphonectria* [30] and the *Heterobasidion RNA virus 1* (HetRV1; family Partitiviridae) in the genus *Heterobasidion* [31]. For HetRV1, Vainio et al. [31] detected high nucleotide level similarity (98%) between HetRV1 obtained from taxonomically distant *H. parviporum* and *H. australe*, thus suggesting a recent HetRV1 transmission in nature. Furthermore, Deng et al. [33] reported a mitovirus to be conspecific in *Sclerotinia homoeocarpa*, the causal agent of Dollar spot, and *Ophiostoma novo-ulmi*, the causal agent of Dutch elm disease, with 92.4% (nucleotide) and 95.1% (amino acid) sequence identities between viral strains and [45] detected 95% identity of BcMV1 and *Ophiostoma novo-ulmi mitovirus 3b*.

In contrast to plant and animal pathogenic viruses, natural vectors are largely unknown in fungal viruses, which typically have no extracellular phase [25]. In vitro experiments (e.g., [30,32]) have shown that mycoviruses are transmitted horizontally between fungal species via hyphal anastomosis. Recent genomic studies revealed that the genomes of *H. fraxineus* and *H. albidus* are highly similar [46] and 75% of the *H. albidus* reads could be mapped to the *H. fraxineus* reference genome [16]. Hence, hyphal anastomosis and transfer of genetic elements between both fungal species without the final hybridization event cannot be excluded. The low detection rate of such events (i.e., intermixing of HfMV1 sequences from *H. fraxineus* with sequence clusters of HfMV1 from *H. albidus*) in this study might be simply due to the fact that it is still too early in time to detect them more frequently. Moreover, it has to be kept in mind, that most likely we did not capture the full picture and all transmission events, as only a small number of *H. albidus* isolates from Switzerland and France were investigated in the present study. Future sampling of *H. fraxineus* in areas where divergent HfMV1 strains were found in *H. albidus* (e.g., Southern Switzerland or North-Western France), as well as the inclusion of additional *H. albidus* samples from other countries could reveal additional evidence of interspecies transmission of HfMV1.

## 5. Conclusions

Our study shows that the mitovirus HfMV1 that was previously identified in the ash dieback pathogen *H. fraxineus* [26] is also occurring in the congeneric species *H. albidus*. Sampling history together with phylogenetic and population genetic analyses suggest that HfMV1 is a native mycovirus in the European *H. albidus* population. The occurrence of a conspecific mitovirus in *H. albidus* and *H. fraxineus* is most likely the result of parallel virus evolution in the two fungal hosts. In addition, our study provides evidence for interspecies virus transmission from *H. albidus* to *H. fraxineus*, which contributed to the viral diversity observed in the invasive *H. fraxineus* population in Europe.

## Figures and Tables

**Table 1:** Overview of the *Hymenoscyphus albidus* and *H. fraxineus* isolates included in the present study.

Host	Region (Abbreviation)	Canton (s)/Region	Number of Samples Screened	Number of Samples with HfMV1	Sampling Year (s)	Reference for Fungal Isolates
*H. albidus*			67	34		
Switzerland	South-Western Switzerland (SW-CH)	VS, VD	13	13	2008, 2009, 2012	This study
	Southern Switzerland (S-CH)	TI	33	12	2009, 2010, 2012	[35]; this study
	Northern Switzerland (N-CH)	GL, ZH	7	4	2009	[35]
France	North-Western France (NW-F)	Bretagne	14	5	2012	[17]; this study
*H. fraxineus*			**	221		
Switzerland	South-Western Switzerland (SW-CH)	VS, VD	**	41	2013, 2016	[13]; this study
	Southern-Switzerland * (S-CH)	TI, GR	**	90	2014, 2013, 2016	[15]; this study
	Northern-Switzerland (N-CH)	SG, SZ, UR	**	85	2013, 2014	[13]; this study

* Including two HfMV1 positive isolates from Northern Italy; ** HfMV1 positive isolates were selected, no data on number initially screened.

**Table 2:** Population genetic parameters for the full length RdRP gene sequences (2151 bp) of HfMV1 recovered from *Hymenoscyphus albidus* and *H. fraxineus*.

Population	N	Sequence Length	Net Sites	S	η	h	Hd	π	K
*H. fraxineus*	32	2151	1791	219	239	30	1.0	0.021	38
*H. albidus*	8	2151	2122	400	464	8	1.0	0.077	163
*H. fraxineus* CH	30	2151	1851	218	238	28	1.0	0.021	39
*H. albidus* CH	6	2151	2129	360	403	6	1.0	0.076	163
*H. fraxineus* & *H. albidus* total	40	2151	1774	328	380	38	1.0	0.029	52

Abbreviations: N, number of isolates; Sequence length, entire sequence length in bp; Net sites, sequence length in analysis; S, number of segregating sites; η, total number of mutations occurred in that population; h, number of haplotypes; Hd, haplotype diversity; π, nucleotide diversity estimated by the average number of differences per site between two sequences; K, average number of nucleotide differences; CH, only samples from Switzerland (30 out of 32 in *H. fraxineus* and 6 out of 8 in *H. albidus*).

**Table 3:** Population genetic parameters for the partial RdRP gene sequences (495 bp) of HfMV1 recovered from *Hymenoscyphus albidus* and *H. fraxineus*.

Population	N	Sequence Length	Net Sites	S	η	h	Hd	π	K
*H. fraxineus* all	221	495	348	49	57	48	0.79	0.007	2.35
*H. fraxineus* CH	214	495	349	50	58	49	0.80	0.007	2.41
*H. fraxineus* S-CH *	90	495	359	33	37	28	0.80	0.008	2.98
*H. fraxineus* SW-CH	41	495	485	89	102	27	0.96	0.039	19.21
*H. fraxineus* N-CH	85	495	458	73	81	53	0.96	0.019	8.72
*H. fraxineus* NW-F	5	495	488	16	16	3	0.70	0.014	6.60
*H. albidus* all	34	495	426	99	129	28	0.99	0.080	34.02
*H. albidus* CH	29	495	427	94	122	24	0.99	0.078	33.39
*H. albidus* S-CH	12	495	443	79	94	12	1.00	0.067	29.74
*H. albidus* SW-CH	13	495	467	76	88	10	0.96	0.052	24.03
*H. albidus* NW-F	5	495	494	34	34	4	0.90	0.026	14.40
*H. fraxineus* & *H. albidus* total	255	495	348	71	89	73	0.84	0.015	5.33

Only populations ≥5 samples were considered. Abbreviations: N, number of isolates; Sequence length, entire sequence length in bp; Net sites, sequence length in analysis; S, number of segregating sites; η, total number of mutations occurred in that population; h, number of haplotypes; Hd, haplotype diversity; π, nucleotide diversity estimated by the average number of differences per site between two sequences; K, average number of nucleotide differences; CH: Switzerland, S-CH: Southern Switzerland, SW-CH: South-Western Switzerland, N-CH: Northern Switzerland, NW-F: North-Western France, * including 2 isolates from Northern Italy.

## Supplementary Materials

The following are available online at http://www.mdpi.com/1999-4915/10/11/628/s1, **Figure S1:** Phylogenetic tree resulting from the RaxML analysis conducted with 40 (8 *H. albidus*, 32 *H. fraxineus*) full sequences (2151 bp) of the RdRP gene of the mitovirus HfMV1. Green color depicts HfMV1 *H. albidus* group isolates. Black color depicts HfMV1 group 2 isolates and grey color HfMV1 group isolates. Blue color and *** mark *H. fraxineus* isolates within the *H. albidus* group. Hf characterizes viral sequences from *H. fraxineus*, Ha from *H. albidus* isolates. For abbreviation see Table 1. Bootstrap values are shown if >70. Arrows indicate intermixing of *H. albidus* and *H. fraxineus* HfMV1 sequences. **Figure S2:** RAxML analysis conducted with 255 sequences (495 bp) of the partial RdRP gene of the mitovirus HfMV1. Each sample is named with the sampling year, followed by the sampling locality (for abbreviation see Table 1) and sample name. Bootstrap values are show if ≥60. Samples marked in bold and italics are included in the full-length analysis. **Table S1:** Details of the isolates in the study with sample locations, years and GenBank accession numbers. **Table S2:** List of additional primers used to sequence the full open reading frame of *Hymenoscyphus fraxineus mitovirus 1* and 5′ and 3′ flanking regions of HfMV1. Tm = annealing temperature in degrees Celsius.
