# Supplementary material for: Detection of a Conspecific Mycovirus in Two Closely Related Native and Introduced Fungal Hosts and Evidence for Interspecific Virus Transmission
Source: Viruses. 2018 Nov 13;10(11):628. doi: 10.3390/v10110628 (PMC6266060; doi:10.3390/v10110628)
Supplement: Supplementary file 1 [file viruses-10-00628-s001.pdf]

Supporting Materials

Manuscript title

Detection of a conspecific mycovirus in two closely related native and introduced fungal hosts and evidence for interspecific virus transmission

Authors

Corine N. Schoebel, Simone Prospero, Andrin Gross, Daniel Rigling
Swiss Federal Institute for Forest, Snow and Landscape Research, WSL, Zuercherstrasse 111, 8903 Birmensdorf, Switzerland.

Table S1: Details on the isolates in the study with sample locations, years and GenBank accession numbers, etc. *In case sequences were previously submitted to GenBank, the respective reverence is given. – indicates that no sequence was submitted.

							GenBank Accession numbers				
ID	Year	Species	Canton	Coordinates N	Coordinates E	Region	RdRP 495 *	RdRP full ORF	ITS_	SSR	Reference
AIG01L	2013	*Hymenoscyphus fraxineus*	Vaud (VD)	46.32589	6.93349	South-Western Switzerland (SW-CH)	[13]		no	x	[13]
AIG01P	2013	*Hymenoscyphus fraxineus*	Vaud (VD)	46.32589	6.93349	South-Western Switzerland (SW-CH)	-	MK079873	no	x	[13]
AIG02L	2013	*Hymenoscyphus fraxineus*	Vaud (VD)	46.32589	6.93349	South-Western Switzerland (SW-CH)	[13]		no	x	[13]
AIG04L	2013	*Hymenoscyphus fraxineus*	Vaud (VD)	46.32589	6.93349	South-Western Switzerland (SW-CH)	[13]		no	x	[13]
AIG06P	2013	*Hymenoscyphus fraxineus*	Vaud (VD)	46.32589	6.93349	South-Western Switzerland (SW-CH)	[13]		no	x	[13]
AIG09P	2013	*Hymenoscyphus fraxineus*	Vaud (VD)	46.32589	6.93349	South-Western Switzerland (SW-CH)	[13]		no	x	[13]
AIG11P	2013	*Hymenoscyphus fraxineus*	Vaud (VD)	46.32589	6.93349	South-Western Switzerland (SW-CH)	[13]		no	x	[13]
AIG16P	2013	*Hymenoscyphus fraxineus*	Vaud (VD)	46.32589	6.93349	South-Western Switzerland (SW-CH)	[13]		no	x	[13]
AIG20L	2013	*Hymenoscyphus fraxineus*	Vaud (VD)	46.32589	6.93349	South-Western Switzerland (SW-CH)	[13]		no	x	[13]
AIG22P	2013	*Hymenoscyphus fraxineus*	Vaud (VD)	46.32589	6.93349	South-Western Switzerland (SW-CH)	[13]		no	x	[13]
AIG23L	2013	*Hymenoscyphus fraxineus*	Vaud (VD)	46.32589	6.93349	South-Western Switzerland (SW-CH)	[13]		no	x	[13]
AIG23P	2013	*Hymenoscyphus fraxineus*	Vaud (VD)	46.32589	6.93349	South-Western Switzerland (SW-CH)	[13]		no	x	[13]
AIG29P	2013	*Hymenoscyphus fraxineus*	Vaud (VD)	46.32589	6.93349	South-Western Switzerland (SW-CH)	-	MK079874	no	x	[13]
AIG35L	2013	*Hymenoscyphus fraxineus*	Vaud (VD)	46.32589	6.93349	South-Western Switzerland (SW-CH)	[13]		no	x	[13]
AIG35P	2013	*Hymenoscyphus fraxineus*	Vaud (VD)	46.32589	6.93349	South-Western Switzerland (SW-CH)	-	MK079876	no	x	[13]
AIG41L	2013	*Hymenoscyphus fraxineus*	Vaud (VD)	46.32589	6.93349	South-Western Switzerland (SW-CH)	[13]		no	x	[13]
AIG43L	2013	*Hymenoscyphus fraxineus*	Vaud (VD)	46.32589	6.93349	South-Western Switzerland (SW-CH)	-	MK079875	no	x	[13]
AIG46P	2013	*Hymenoscyphus fraxineus*	Vaud (VD)	46.32589	6.93349	South-Western Switzerland (SW-CH)	[13]		no	x	[13]
AIG47L	2013	*Hymenoscyphus fraxineus*	Vaud (VD)	46.53104	6.36983	South-Western Switzerland (SW-CH)	-	In revision	no	x	[13]
AIG60L	2013	*Hymenoscyphus fraxineus*	Vaud (VD)	46.32589	6.93349	South-Western Switzerland (SW-CH)	[13]		no	x	[13]
BIE05L	2013	*Hymenoscyphus fraxineus*	Vaud (VD)	46.53104	6.36983	South-Western Switzerland (SW-CH)	[13]		no	x	[13]
BIE14L	2013	*Hymenoscyphus fraxineus*	Vaud (VD)	46.53104	6.36983	South-Western Switzerland (SW-CH)	-	MK079877	no	x	[13]
BIE16L	2013	*Hymenoscyphus fraxineus*	Vaud (VD)	46.53104	6.36983	South-Western Switzerland (SW-CH)	[13]		no	x	[13]
BIE17P	2013	*Hymenoscyphus fraxineus*	Vaud (VD)	46.53104	6.36983	South-Western Switzerland (SW-CH)	[13]		no	x	[13]
BIE21P	2013	*Hymenoscyphus fraxineus*	Vaud (VD)	46.32589	6.93349	South-Western Switzerland (SW-CH)	-	MK079878	no	x	[13]
BIE22L	2013	*Hymenoscyphus fraxineus*	Vaud (VD)	46.53104	6.36983	South-Western Switzerland (SW-CH)	-	MK079879	no	x	[13]
BIE24L	2013	*Hymenoscyphus fraxineus*	Vaud (VD)	46.53104	6.36983	South-Western Switzerland (SW-CH)	-	MK079880	no	x	[13]
BIE25L	2013	*Hymenoscyphus fraxineus*	Vaud (VD)	46.53104	6.36983	South-Western Switzerland (SW-CH)	-	MK079881	no	x	[13]
BIE26P	2013	*Hymenoscyphus fraxineus*	Vaud (VD)	46.53104	6.36983	South-Western Switzerland (SW-CH)	[13]		no	x	[13]
BIE31P	2013	*Hymenoscyphus fraxineus*	Vaud (VD)	46.53104	6.36983	South-Western Switzerland (SW-CH)	[13]		no	x	[13]
BIE32P	2013	*Hymenoscyphus fraxineus*	Vaud (VD)	46.53104	6.36983	South-Western Switzerland (SW-CH)	-	MK079882	no	x	[13]
BIE39L	2013	*Hymenoscyphus fraxineus*	Vaud (VD)	46.53104	6.36983	South-Western Switzerland (SW-CH)	[13]		no	x	[13]
BIE43P	2013	*Hymenoscyphus fraxineus*	Vaud (VD)	46.53104	6.36983	South-Western Switzerland (SW-CH)	[13]		no	x	[13]
BIE45P	2013	*Hymenoscyphus fraxineus*	Vaud (VD)	46.53104	6.36983	South-Western Switzerland (SW-CH)	[13]		no	x	[13]
BIE48L	2013	*Hymenoscyphus fraxineus*	Vaud (VD)	46.53104	6.36983	South-Western Switzerland (SW-CH)	[13]		no	x	[13]
BIE49P	2013	*Hymenoscyphus fraxineus*	Vaud (VD)	46.53104	6.36983	South-Western Switzerland (SW-CH)	[13]		no	x	[13]
BIE53L	2013	*Hymenoscyphus fraxineus*	Vaud (VD)	46.53104	6.36983	South-Western Switzerland (SW-CH)	[13]		no	x	[13]
BIE54P	2013	*Hymenoscyphus fraxineus*	Vaud (VD)	46.53104	6.36983	South-Western Switzerland (SW-CH)	[13]		no	x	[13]
BIE57L	2013	*Hymenoscyphus fraxineus*	Vaud (VD)	46.53104	6.36983	South-Western Switzerland (SW-CH)	[13]		no	x	[13]
BIE59P	2013	*Hymenoscyphus fraxineus*	Vaud (VD)	46.53104	6.36983	South-Western Switzerland (SW-CH)	[13]		no	x	[13]
KUE02L	2013	*Hymenoscyphus fraxineus*	Schwyz (SZ)	47.07013	8.458	Northern Switzerland (N-CH)	[13]		no	x	[13]
KUE05L	2013	*Hymenoscyphus fraxineus*	Schwyz (SZ)	47.07013	8.458	Northern Switzerland (N-CH)	-	MK079872	no	x	[13]
KUE06L	2013	*Hymenoscyphus fraxineus*	Schwyz (SZ)	47.07013	8.458	Northern Switzerland (N-CH)	[13]		no	x	[13]
KUE06P	2013	*Hymenoscyphus fraxineus*	Schwyz (SZ)	47.07013	8.458	Northern Switzerland (N-CH)	[13]		no	x	[13]
KUE10L	2013	*Hymenoscyphus fraxineus*	Schwyz (SZ)	47.07013	8.458	Northern Switzerland (N-CH)	-	MK079869	no	x	[13]
KUE11L	2013	*Hymenoscyphus fraxineus*	Schwyz (SZ)	47.07013	8.458	Northern Switzerland (N-CH)	[13]		no	x	[13]
KUE11P	2013	*Hymenoscyphus fraxineus*	Schwyz (SZ)	47.07013	8.458	Northern Switzerland (N-CH)	[13]		no	x	[13]
KUE24L	2013	*Hymenoscyphus fraxineus*	Schwyz (SZ)	47.07013	8.458	Northern Switzerland (N-CH)	-	MK079870	no	x	[13]
KUE13L	2013	*Hymenoscyphus fraxineus*	Schwyz (SZ)	47.07013	8.458	Northern Switzerland (N-CH)	[13]		no	x	[13]
KUE15L	2013	*Hymenoscyphus fraxineus*	Schwyz (SZ)	47.07013	8.458	Northern Switzerland (N-CH)	[13]		no	x	[13]
KUE24P	2013	*Hymenoscyphus fraxineus*	Schwyz (SZ)	47.07013	8.458	Northern Switzerland (N-CH)	[13]		no	x	[13]
KUE36L	2013	*Hymenoscyphus fraxineus*	Schwyz (SZ)	47.07013	8.458	Northern Switzerland (N-CH)	[13]		no	x	[13]
KUE37P	2013	*Hymenoscyphus fraxineus*	Schwyz (SZ)	47.07013	8.458	Northern Switzerland (N-CH)	-	MK079871	no	x	[13]
KUE39P	2013	*Hymenoscyphus fraxineus*	Schwyz (SZ)	47.07013	8.458	Northern Switzerland (N-CH)	[13]		no	x	[13]
KUE42P	2013	*Hymenoscyphus fraxineus*	Schwyz (SZ)	47.07013	8.458	Northern Switzerland (N-CH)	[13]		no	x	[13]

KUE43P	2013	*Hymenoscyphus fraxineus*	Schwyz (SZ)	47.07013	8.458	Northern Switzerland (N-CH)	[13]		no	x	[13]
KUE53L	2013	*Hymenoscyphus fraxineus*	Schwyz (SZ)	47.07013	8.458	Northern Switzerland (N-CH)	[13]		no	x	[13]
KUE53P	2013	*Hymenoscyphus fraxineus*	Schwyz (SZ)	47.07013	8.458	Northern Switzerland (N-CH)	[13]		no	x	[13]
KUE54P	2013	*Hymenoscyphus fraxineus*	Schwyz (SZ)	47.07013	8.458	Northern Switzerland (N-CH)	[13]		no	x	[13]
KUE60P	2013	*Hymenoscyphus fraxineus*	Schwyz (SZ)	47.07013	8.458	Northern Switzerland (N-CH)	[13]		no	x	[13]
SAR02P	2013	*Hymenoscyphus fraxineus*	St Gallen (SG)	47.26583	9.48278	Northern Switzerland (N-CH)	[13]		no	x	[13]
SAR03L	2013	*Hymenoscyphus fraxineus*	St Gallen (SG)	47.26583	9.48278	Northern Switzerland (N-CH)	-	MK079859	no	x	[13]
SAR07L	2013	*Hymenoscyphus fraxineus*	St Gallen (SG)	47.26583	9.48278	Northern Switzerland (N-CH)	[13]		no	x	[13]
SAR07P	2013	*Hymenoscyphus fraxineus*	St Gallen (SG)	47.26583	9.48278	Northern Switzerland (N-CH)	[13]		no	x	[13]
SAR11P	2013	*Hymenoscyphus fraxineus*	St Gallen (SG)	47.26583	9.48278	Northern Switzerland (N-CH)	[13]		no	x	[13]
SAR12L	2013	*Hymenoscyphus fraxineus*	St Gallen (SG)	47.26583	9.48278	Northern Switzerland (N-CH)	[13]		no	x	[13]
SAR13L	2013	*Hymenoscyphus fraxineus*	St Gallen (SG)	47.26583	9.48278	Northern Switzerland (N-CH)	-	MK079855	no	x	[13]
SAR14P	2013	*Hymenoscyphus fraxineus*	St Gallen (SG)	47.26583	9.48278	Northern Switzerland (N-CH)	[13]		no	x	[13]
SAR16L	2013	*Hymenoscyphus fraxineus*	St Gallen (SG)	47.26583	9.48278	Northern Switzerland (N-CH)	[13]		no	x	[13]
SAR22L	2013	*Hymenoscyphus fraxineus*	St Gallen (SG)	47.26583	9.48278	Northern Switzerland (N-CH)	[13]		no	x	[13]
SAR24L	2013	*Hymenoscyphus fraxineus*	St Gallen (SG)	47.26583	9.48278	Northern Switzerland (N-CH)	-	MK079856	no	x	[13]
SAR26P	2013	*Hymenoscyphus fraxineus*	St Gallen (SG)	47.26583	9.48278	Northern Switzerland (N-CH)	-	MK079857	no	x	[13]
SAR28L	2013	*Hymenoscyphus fraxineus*	St Gallen (SG)	47.26583	9.48278	Northern Switzerland (N-CH)	-	MK079858	no	x	[13]
SAR28P	2013	*Hymenoscyphus fraxineus*	St Gallen (SG)	47.26583	9.48278	Northern Switzerland (N-CH)	[13]		no	x	[13]
SAR33L	2013	*Hymenoscyphus fraxineus*	St Gallen (SG)	47.26583	9.48278	Northern Switzerland (N-CH)	[13]		no	x	[13]
SAR41P	2013	*Hymenoscyphus fraxineus*	St Gallen (SG)	47.26583	9.48278	Northern Switzerland (N-CH)	[13]		no	x	[13]
SAR43P	2013	*Hymenoscyphus fraxineus*	St Gallen (SG)	47.26583	9.48278	Northern Switzerland (N-CH)	-	MK079860	no	x	[13]
SAR48L	2013	*Hymenoscyphus fraxineus*	St Gallen (SG)	47.26583	9.48278	Northern Switzerland (N-CH)	[13]		no	x	[13]
SAR51L	2013	*Hymenoscyphus fraxineus*	St Gallen (SG)	47.26583	9.48278	Northern Switzerland (N-CH)	-	MK079861	no	x	[13]
SAR52P	2013	*Hymenoscyphus fraxineus*	St Gallen (SG)	47.26583	9.48278	Northern Switzerland (N-CH)	[13]		no	x	[13]
WIL03L	2013	*Hymenoscyphus fraxineus*	St Gallen (SG)	47.50861	9.155	Northern Switzerland (N-CH)	[13]		no	x	[13]
WIL06P	2013	*Hymenoscyphus fraxineus*	St Gallen (SG)	47.50861	9.155	Northern Switzerland (N-CH)	[13]		no	x	[13]
WIL10L	2013	*Hymenoscyphus fraxineus*	St Gallen (SG)	47.50861	9.155	Northern Switzerland (N-CH)	[13]		no	x	[13]
WIL12P	2013	*Hymenoscyphus fraxineus*	St Gallen (SG)	47.50861	9.155	Northern Switzerland (N-CH)	[13]		no	x	[13]
WIL13L	2013	*Hymenoscyphus fraxineus*	St Gallen (SG)	47.50861	9.155	Northern Switzerland (N-CH)	[13]		no	x	[13]
WIL18L	2013	*Hymenoscyphus fraxineus*	St Gallen (SG)	47.50861	9.155	Northern Switzerland (N-CH)	[13]		no	x	[13]
WIL19L	2013	*Hymenoscyphus fraxineus*	St Gallen (SG)	47.50861	9.155	Northern Switzerland (N-CH)	-	MK079862	no	x	[13]
WIL19P	2013	*Hymenoscyphus fraxineus*	St Gallen (SG)	47.50861	9.155	Northern Switzerland (N-CH)	[13]		no	x	[13]
WIL20L	2013	*Hymenoscyphus fraxineus*	St Gallen (SG)	47.50861	9.155	Northern Switzerland (N-CH)	-	MK079863	no	x	[13]
WIL22L	2013	*Hymenoscyphus fraxineus*	St Gallen (SG)	47.50861	9.155	Northern Switzerland (N-CH)	-	MK079864	no	x	[13]
WIL22P	2013	*Hymenoscyphus fraxineus*	St Gallen (SG)	47.50861	9.155	Northern Switzerland (N-CH)	[13]		no	x	[13]
WIL27P	2013	*Hymenoscyphus fraxineus*	St Gallen (SG)	47.50861	9.155	Northern Switzerland (N-CH)	[13]		no	x	[13]
WIL32P	2013	*Hymenoscyphus fraxineus*	St Gallen (SG)	47.50861	9.155	Northern Switzerland (N-CH)	[13]		no	x	[13]
WIL34P	2013	*Hymenoscyphus fraxineus*	St Gallen (SG)	47.50861	9.155	Northern Switzerland (N-CH)	[13]		no	x	[13]
WIL37P	2013	*Hymenoscyphus fraxineus*	St Gallen (SG)	47.50861	9.155	Northern Switzerland (N-CH)	-	MK079865	no	x	[13]
WIL41P	2013	*Hymenoscyphus fraxineus*	St Gallen (SG)	47.50861	9.155	Northern Switzerland (N-CH)	[13]		no	x	[13]
WIL47L	2013	*Hymenoscyphus fraxineus*	St Gallen (SG)	47.50861	9.155	Northern Switzerland (N-CH)	-	MK079866	no	x	[13]
WIL49L	2013	*Hymenoscyphus fraxineus*	St Gallen (SG)	47.50861	9.155	Northern Switzerland (N-CH)	[13]		no	x	[13]
WIL54P	2013	*Hymenoscyphus fraxineus*	St Gallen (SG)	47.50861	9.155	Northern Switzerland (N-CH)	-	MK079867	no	x	[13]
WIL56L	2013	*Hymenoscyphus fraxineus*	St Gallen (SG)	47.50861	9.155	Northern Switzerland (N-CH)	-	MK079868	no	x	[13]
GSE1	2014	*Hymenoscyphus fraxineus*	Ile de France	48.4678	2.3532	North-Western France (France)	[15]		no	x	[15]
GSE2	2014	*Hymenoscyphus fraxineus*	Ile de France	48.4678	2.3532	North-Western France (France)	[15]		no	x	[15]
GSE6	2014	*Hymenoscyphus fraxineus*	Ile de France	48.4678	2.3532	North-Western France (France)	[15]		no	x	[15]
NSE2	2014	*Hymenoscyphus fraxineus*	Ile de France	48.3683	2.5083	North-Western France (France)	[15]		no	x	[15]
NSE8	2014	*Hymenoscyphus fraxineus*	Ile de France	48.3683	2.5083	North-Western France (France)	[15]		no	x	[15]
VIC1	2014	*Hymenoscyphus fraxineus*	Dolomites	46.416667	11.666667	Southern Switzerland (S-CH)	[15]		no	x	[15]
VIC2	2014	*Hymenoscyphus fraxineus*	Dolomites	46.416667	11.666667	Southern Switzerland (S-CH)	[15]		no	x	[15]
CEV1	2014	*Hymenoscyphus fraxineus*	Ticino (TI)	46.31112	8.60697	Southern Switzerland (S-CH)	-	MK079883			this study
CEV3	2014	*Hymenoscyphus fraxineus*	Ticino (TI)	46.31112	8.60697	Southern Switzerland (S-CH)	MK079718				this study
FOR2	2014	*Hymenoscyphus fraxineus*	Ticino (TI)	46.37395	8.54773	Southern Switzerland (S-CH)	MK079719				this study
FOR20	2014	*Hymenoscyphus fraxineus*	Ticino (TI)	46.37395	8.54773	Southern Switzerland (S-CH)	MK079720				this study
FOR28	2014	*Hymenoscyphus fraxineus*	Ticino (TI)	46.37395	8.54773	Southern Switzerland (S-CH)	MK079721				this study
FOR5	2014	*Hymenoscyphus fraxineus*	Ticino (TI)	46.37395	8.54773	Southern Switzerland (S-CH)	MK079722				this study
FOR6	2014	*Hymenoscyphus fraxineus*	Ticino (TI)	46.37395	8.54773	Southern Switzerland (S-CH)	MK079723				this study
FORdr	2014	*Hymenoscyphus fraxineus*	Ticino (TI)	46.37395	8.54773	Southern Switzerland (S-CH)	MK079724				this study
RIV16	2014	*Hymenoscyphus fraxineus*	Ticino (TI)	46.29343	8.63958	Southern Switzerland (S-CH)	MK079725				this study

RIV17	2014	*Hymenoscyphus fraxineus*	Ticino (TI)	46.29343	8.63958	Southern Switzerland (S-CH)	MK079726	this study
RIV18	2014	*Hymenoscyphus fraxineus*	Ticino (TI)	46.29343	8.63958	Southern Switzerland (S-CH)	MK079727	this study
RIV19	2014	*Hymenoscyphus fraxineus*	Ticino (TI)	46.29343	8.63958	Southern Switzerland (S-CH)	MK079728	this study
RIV20	2014	*Hymenoscyphus fraxineus*	Ticino (TI)	46.29343	8.63958	Southern Switzerland (S-CH)	MK079729	this study
RIV21	2014	*Hymenoscyphus fraxineus*	Ticino (TI)	46.29343	8.63958	Southern Switzerland (S-CH)	-	this study
RIV22	2014	*Hymenoscyphus fraxineus*	Ticino (TI)	46.29343	8.63958	Southern Switzerland (S-CH)	MK079730	this study
RIV23	2014	*Hymenoscyphus fraxineus*	Ticino (TI)	46.29343	8.63958	Southern Switzerland (S-CH)	MK079731	this study
RIV24	2014	*Hymenoscyphus fraxineus*	Ticino (TI)	46.29343	8.63958	Southern Switzerland (S-CH)	MK079732	this study
RIV25	2014	*Hymenoscyphus fraxineus*	Ticino (TI)	46.29343	8.63958	Southern Switzerland (S-CH)	MK079733	this study
RIV27	2014	*Hymenoscyphus fraxineus*	Ticino (TI)	46.29343	8.63958	Southern Switzerland (S-CH)	MK079734	this study
RIV28	2014	*Hymenoscyphus fraxineus*	Ticino (TI)	46.29343	8.63958	Southern Switzerland (S-CH)	MK079735	this study
RIV29	2014	*Hymenoscyphus fraxineus*	Ticino (TI)	46.29343	8.63958	Southern Switzerland (S-CH)	MK079736	this study
RIVdr	2014	*Hymenoscyphus fraxineus*	Ticino (TI)	46.29343	8.63958	Southern Switzerland (S-CH)	MK079737	this study
ROD12	2014	*Hymenoscyphus fraxineus*	Ticino (TI)	46.49869	8.72837	Southern Switzerland (S-CH)	MK079738	this study
ROD14	2014	*Hymenoscyphus fraxineus*	Ticino (TI)	46.49869	8.72837	Southern Switzerland (S-CH)	MK079739	this study
ROD15	2014	*Hymenoscyphus fraxineus*	Ticino (TI)	46.49869	8.72837	Southern Switzerland (S-CH)	MK079740	this study
ROD16	2014	*Hymenoscyphus fraxineus*	Ticino (TI)	46.49869	8.72837	Southern Switzerland (S-CH)	MK079741	this study
ROD18	2014	*Hymenoscyphus fraxineus*	Ticino (TI)	46.49869	8.72837	Southern Switzerland (S-CH)	MK079742	this study
ROD19	2014	*Hymenoscyphus fraxineus*	Ticino (TI)	46.49869	8.72837	Southern Switzerland (S-CH)	MK079743	this study
ROD20	2014	*Hymenoscyphus fraxineus*	Ticino (TI)	46.49869	8.72837	Southern Switzerland (S-CH)	MK079744	this study
ROD21	2014	*Hymenoscyphus fraxineus*	Ticino (TI)	46.49869	8.72837	Southern Switzerland (S-CH)	-	this study
ROD24	2014	*Hymenoscyphus fraxineus*	Ticino (TI)	46.49869	8.72837	Southern Switzerland (S-CH)	MK079745	this study
ROD25	2014	*Hymenoscyphus fraxineus*	Ticino (TI)	46.49869	8.72837	Southern Switzerland (S-CH)	-	this study
ROD26	2014	*Hymenoscyphus fraxineus*	Ticino (TI)	46.49869	8.72837	Southern Switzerland (S-CH)	MK079746	this study
ROD27	2014	*Hymenoscyphus fraxineus*	Ticino (TI)	46.49869	8.72837	Southern Switzerland (S-CH)	MK079747	this study
ROD28	2014	*Hymenoscyphus fraxineus*	Ticino (TI)	46.49869	8.72837	Southern Switzerland (S-CH)	MK079748	this study
ROD29	2014	*Hymenoscyphus fraxineus*	Ticino (TI)	46.49869	8.72837	Southern Switzerland (S-CH)	-	this study
ROD5	2014	*Hymenoscyphus fraxineus*	Ticino (TI)	46.49869	8.72837	Southern Switzerland (S-CH)	-	this study
ROD6	2014	*Hymenoscyphus fraxineus*	Ticino (TI)	46.49869	8.72837	Southern Switzerland (S-CH)	MK079749	this study
ROD7	2014	*Hymenoscyphus fraxineus*	Ticino (TI)	46.49869	8.72837	Southern Switzerland (S-CH)	MK079750	this study
COG12	2014	*Hymenoscyphus fraxineus*	Ticino (TI)	46.26838	8.68245	Southern Switzerland (S-CH)	MK079751	this study
COG13	2014	*Hymenoscyphus fraxineus*	Ticino (TI)	46.26838	8.68245	Southern Switzerland (S-CH)	MK079752	this study
MAG6	2014	*Hymenoscyphus fraxineus*	Ticino (TI)	46.25264	8.69938	Southern Switzerland (S-CH)	MK079753	this study
MAG7a	2014	*Hymenoscyphus fraxineus*	Ticino (TI)	46.25264	8.69938	Southern Switzerland (S-CH)	MK079754	this study
MAG7b	2014	*Hymenoscyphus fraxineus*	Ticino (TI)	46.25264	8.69938	Southern Switzerland (S-CH)	MK079755	this study
SCAR17	2014	*Hymenoscyphus fraxineus*	Ticino (TI)	46.41017	8.52587	Southern Switzerland (S-CH)	MK079756	this study
SCAR21	2014	*Hymenoscyphus fraxineus*	Ticino (TI)	46.41017	8.52587	Southern Switzerland (S-CH)	MK079757	this study
SCAR26	2014	*Hymenoscyphus fraxineus*	Ticino (TI)	46.41017	8.52587	Southern Switzerland (S-CH)	MK079758	this study
WAS10	2014	*Hymenoscyphus fraxineus*	Uri (UR)	46.70185	8.59726	Northern Switzerland (N-CH)	MK079759	this study
WAS11	2014	*Hymenoscyphus fraxineus*	Uri (UR)	46.70185	8.59726	Northern Switzerland (N-CH)	MK079760	this study
WAS12	2014	*Hymenoscyphus fraxineus*	Uri (UR)	46.70185	8.59726	Northern Switzerland (N-CH)	MK079761	this study
WAS14	2014	*Hymenoscyphus fraxineus*	Uri (UR)	46.70185	8.59726	Northern Switzerland (N-CH)	MK079762	this study
WAS15	2014	*Hymenoscyphus fraxineus*	Uri (UR)	46.70185	8.59726	Northern Switzerland (N-CH)	MK079763	this study
WAS16	2014	*Hymenoscyphus fraxineus*	Uri (UR)	46.70185	8.59726	Northern Switzerland (N-CH)	MK079764	this study
WAS17	2014	*Hymenoscyphus fraxineus*	Uri (UR)	46.70185	8.59726	Northern Switzerland (N-CH)	-	this study
WAS18	2014	*Hymenoscyphus fraxineus*	Uri (UR)	46.70185	8.59726	Northern Switzerland (N-CH)	MK079765	this study
WAS19a	2014	*Hymenoscyphus fraxineus*	Uri (UR)	46.70185	8.59726	Northern Switzerland (N-CH)	MK079766	this study
WAS19b	2014	*Hymenoscyphus fraxineus*	Uri (UR)	46.70185	8.59726	Northern Switzerland (N-CH)	MK079767	this study
WAS20	2014	*Hymenoscyphus fraxineus*	Uri (UR)	46.70185	8.59726	Northern Switzerland (N-CH)	MK079768	this study
WAS21	2014	*Hymenoscyphus fraxineus*	Uri (UR)	46.70185	8.59726	Northern Switzerland (N-CH)	MK079769	this study
WAS23	2014	*Hymenoscyphus fraxineus*	Uri (UR)	46.70185	8.59726	Northern Switzerland (N-CH)	MK079770	this study
WAS24	2014	*Hymenoscyphus fraxineus*	Uri (UR)	46.70185	8.59726	Northern Switzerland (N-CH)	-	this study
WAS25	2014	*Hymenoscyphus fraxineus*	Uri (UR)	46.70185	8.59726	Northern Switzerland (N-CH)	MK079771	this study
WAS27	2014	*Hymenoscyphus fraxineus*	Uri (UR)	46.70185	8.59726	Northern Switzerland (N-CH)	MK079772	this study
WAS28	2014	*Hymenoscyphus fraxineus*	Uri (UR)	46.70185	8.59726	Northern Switzerland (N-CH)	MK079773	this study
WAS29	2014	*Hymenoscyphus fraxineus*	Uri (UR)	46.70185	8.59726	Northern Switzerland (N-CH)	MK079774	this study
WAS30	2014	*Hymenoscyphus fraxineus*	Uri (UR)	46.70185	8.59726	Northern Switzerland (N-CH)	MK079775	this study
WAS4	2014	*Hymenoscyphus fraxineus*	Uri (UR)	46.70185	8.59726	Northern Switzerland (N-CH)	MK079776	this study
WAS5	2014	*Hymenoscyphus fraxineus*	Uri (UR)	46.70185	8.59726	Northern Switzerland (N-CH)	MK079777	this study
WAS6	2014	*Hymenoscyphus fraxineus*	Uri (UR)	46.70185	8.59726	Northern Switzerland (N-CH)	MK079778	this study
WAS7	2014	*Hymenoscyphus fraxineus*	Uri (UR)	46.70185	8.59726	Northern Switzerland (N-CH)	MK079779	this study

WAS8	2014	*Hymenoscyphus fraxineus*	Uri (UR)	46.70185	8.59726	Northern Switzerland (N-CH)	MK079780			this study
WAS9	2014	*Hymenoscyphus fraxineus*	Uri (UR)	46.70185	8.59726	Northern Switzerland (N-CH)	-			this study
SOA10c	2016	*Hymenoscyphus fraxineus*	Grison (GR)	46.35	9.216667	Southern Switzerland (S-CH)	MK079714		-	this study
SOA2a	2016	*Hymenoscyphus fraxineus*	Grison (GR)	46.35	9.216667	Southern Switzerland (S-CH)	MK079715		-	this study
SOA8a	2016	*Hymenoscyphus fraxineus*	Grison (GR)	46.35	9.216667	Southern Switzerland (S-CH)	MK079716		-	this study
SOA9b	2016	*Hymenoscyphus fraxineus*	Grison (GR)	46.35	9.216667	Southern Switzerland (S-CH)	MK079717		-	this study
COR10a	2016	*Hymenoscyphus fraxineus*	Ticino (TI)	46.44526	8.93785	Southern Switzerland (S-CH)	MK079781		MF765422	this study
COR1a	2016	*Hymenoscyphus fraxineus*	Ticino (TI)	46.44526	8.93785	Southern Switzerland (S-CH)	MK079782		MF765423	this study
COR3a	2016	*Hymenoscyphus fraxineus*	Ticino (TI)	46.44526	8.93785	Southern Switzerland (S-CH)	-		MF765425	this study
COR5a	2016	*Hymenoscyphus fraxineus*	Ticino (TI)	46.44526	8.93785	Southern Switzerland (S-CH)	MK079783		MF765427	this study
COR6a	2016	*Hymenoscyphus fraxineus*	Ticino (TI)	46.44526	8.93785	Southern Switzerland (S-CH)	-		MF765428	this study
COR7b	2016	*Hymenoscyphus fraxineus*	Ticino (TI)	46.44526	8.93785	Southern Switzerland (S-CH)	MK079784		MF765429	this study
COR8a	2016	*Hymenoscyphus fraxineus*	Ticino (TI)	46.44526	8.93785	Southern Switzerland (S-CH)	MK079785		MF765430	this study
COR9b	2016	*Hymenoscyphus fraxineus*	Ticino (TI)	46.44526	8.93785	Southern Switzerland (S-CH)	MK079786		MF765431	this study
BRI10a	2016	*Hymenoscyphus fraxineus*	Ticino (TI)	46298265	8790192	Southern Switzerland (S-CH)	MK079787			this study
BRI3a	2016	*Hymenoscyphus fraxineus*	Ticino (TI)	46298265	8790192	Southern Switzerland (S-CH)	MK079788			this study
BRI4a	2016	*Hymenoscyphus fraxineus*	Ticino (TI)	46298265	8790192	Southern Switzerland (S-CH)	MK079789			this study
BRI5a	2016	*Hymenoscyphus fraxineus*	Ticino (TI)	46298265	8790192	Southern Switzerland (S-CH)	MK079790			this study
BRI6a	2016	*Hymenoscyphus fraxineus*	Ticino (TI)	46298265	8790192	Southern Switzerland (S-CH)	MK079791			this study
BRI8a	2016	*Hymenoscyphus fraxineus*	Ticino (TI)	46298265	8790192	Southern Switzerland (S-CH)	MK079792			this study
BRI9a	2016	*Hymenoscyphus fraxineus*	Ticino (TI)	46298265	8790192	Southern Switzerland (S-CH)	MK079793			this study
AMB10a	2016	*Hymenoscyphus fraxineus*	Ticino (TI)	46500727	8720870	Southern Switzerland (S-CH)	MK079794			this study
AMB11a	2016	*Hymenoscyphus fraxineus*	Ticino (TI)	46500727	8720870	Southern Switzerland (S-CH)	MK079795			this study
AMB13a	2016	*Hymenoscyphus fraxineus*	Ticino (TI)	46500727	8720870	Southern Switzerland (S-CH)	MK079796			this study
AMB2b	2016	*Hymenoscyphus fraxineus*	Ticino (TI)	46500727	8720870	Southern Switzerland (S-CH)	MK079797			this study
AMB4b	2016	*Hymenoscyphus fraxineus*	Ticino (TI)	46500727	8720870	Southern Switzerland (S-CH)	MK079798			this study
AMB7b	2016	*Hymenoscyphus fraxineus*	Ticino (TI)	46500727	8720870	Southern Switzerland (S-CH)	MK079799			this study
AMB8c	2016	*Hymenoscyphus fraxineus*	Ticino (TI)	46500727	8720870	Southern Switzerland (S-CH)	MK079800			this study
AMB9c	2016	*Hymenoscyphus fraxineus*	Ticino (TI)	46500727	8720870	Southern Switzerland (S-CH)	MK079801			this study
SER13a	2016	*Hymenoscyphus fraxineus*	Ticino (TI)	46.416667	8.983333	Southern Switzerland (S-CH)	-	MK079884		this study
SER14a	2016	*Hymenoscyphus fraxineus*	Ticino (TI)	46.416667	8.983333	Southern Switzerland (S-CH)	MK079802			this study
SER15a	2016	*Hymenoscyphus fraxineus*	Ticino (TI)	46.416667	8.983333	Southern Switzerland (S-CH)	MK079803			this study
SER17b	2016	*Hymenoscyphus fraxineus*	Ticino (TI)	46.416667	8.983333	Southern Switzerland (S-CH)	MK079804			this study
SER18c	2016	*Hymenoscyphus fraxineus*	Ticino (TI)	46.416667	8.983333	Southern Switzerland (S-CH)	MK079805			this study
SER1a	2016	*Hymenoscyphus fraxineus*	Ticino (TI)	46.416667	8.983333	Southern Switzerland (S-CH)	MK079806			this study
SER2c	2016	*Hymenoscyphus fraxineus*	Ticino (TI)	46.416667	8.983333	Southern Switzerland (S-CH)	MK079807			this study
SER3a	2016	*Hymenoscyphus fraxineus*	Ticino (TI)	46.416667	8.983333	Southern Switzerland (S-CH)	MK079808			this study
SER4a	2016	*Hymenoscyphus fraxineus*	Ticino (TI)	46.416667	8.983333	Southern Switzerland (S-CH)	MK079809			this study
SER5a	2016	*Hymenoscyphus fraxineus*	Ticino (TI)	46.416667	8.983333	Southern Switzerland (S-CH)	MK079810			this study
SER6a	2016	*Hymenoscyphus fraxineus*	Ticino (TI)	46.416667	8.983333	Southern Switzerland (S-CH)	MK079811			this study
SER7a	2016	*Hymenoscyphus fraxineus*	Ticino (TI)	46.416667	8.983333	Southern Switzerland (S-CH)	MK079812			this study
SER8c	2016	*Hymenoscyphus fraxineus*	Ticino (TI)	46.416667	8.983333	Southern Switzerland (S-CH)	MK079813			this study
SER9b	2016	*Hymenoscyphus fraxineus*	Ticino (TI)	46.416667	8.983333	Southern Switzerland (S-CH)	MK079814			this study
VIS7a	2016	*Hymenoscyphus fraxineus*	Valais (VS)	46.292216	7.882776	South-Western Switzerland (SW-CH)	MK079713			this study
2373	2009	*Hymenoscyphus albidus*	Valais (VS)	46.22553	7.37201	South-Western Switzerland (SW-CH)	MK079831	MK079852	MF765342	this study
3210	2010	*Hymenoscyphus albidus*	Ticino (TI)	46.154458	8.865217	Southern Switzerland (S-CH)	MK079824		-	[35]; this study
4301	2009	*Hymenoscyphus albidus*	Valais (VS)	46.34	7.633	South-Western Switzerland (SW-CH)	MK079832	MK079854	MF765397	[35]; this study
4310	2009	*Hymenoscyphus albidus*	Ticino (TI)	46.28244	8.56191	Southern Switzerland (S-CH)	MK079819		MF765346	[35]; this study
4312	2009	*Hymenoscyphus albidus*	Glarus (GL)	47.0235	9.04468	Northern Switzerland (N-CH)	MK079816		MF765347	[35]; this study
4315	2009	*Hymenoscyphus albidus*	Glarus (GL)	47.0235	9.04468	Northern Switzerland (N-CH)	MK079817		MF765348	[35]; this study
4324	2009	*Hymenoscyphus albidus*	Glarus (GL)	47.0235	9.04468	Northern Switzerland (N-CH)	MK079818	MK079849	MF765350	[35]; this study
4333	2009	*Hymenoscyphus albidus*	Valais (VS)	46.22553	7.633	South-Western Switzerland (SW-CH)	MK079833		MF765351	[35]; this study
4338	2009	*Hymenoscyphus albidus*	Ticino (TI)	46.26153	8.50237	Southern Switzerland (S-CH)	-		MF765353	[35]; this study
4341	2009	*Hymenoscyphus albidus*	Valais (VS)	46.22553	7.633	South-Western Switzerland (SW-CH)	MK079834		MF765354	[35]; this study
4342	2009	*Hymenoscyphus albidus*	Valais (VS)	46.22553	7.633	South-Western Switzerland (SW-CH)	MK079835		-	[35]; this study
4344	2009	*Hymenoscyphus albidus*	Valais (VS)	46.22553	7.633	South-Western Switzerland (SW-CH)	MK079836		MF765355	[35]; this study
4346	2009	*Hymenoscyphus albidus*	Valais (VS)	46.22553	7.633	South-Western Switzerland (SW-CH)	MK079837		MF765356	[35]; this study
4348	2009	*Hymenoscyphus albidus*	Valais (VS)	46.22553	7.633	South-Western Switzerland (SW-CH)	MK079838		MF765357	[35]; this study
4352	2009	*Hymenoscyphus albidus*	Valais (VS)	46.22553	7.633	South-Western Switzerland (SW-CH)	MK079839	MK079853	-	[35]; this study
4356	2009	*Hymenoscyphus albidus*	Valais (VS)	46.22553	7.633	South-Western Switzerland (SW-CH)	MK079840		MF765358	[35]; this study
4365	2009	*Hymenoscyphus albidus*	Ticino (TI)	46.30148	8.42595	Southern Switzerland (S-CH)	MK079823		MF765361	[35]; this study

4370	2009	*Hymenoscyphus albidus*	Ticino (TI)	46.11532	8.34095	Southern Switzerland (S-CH)	-		-	[35]; this study
4377	2009	*Hymenoscyphus albidus*	Valais (VS)	46.22553	7.633	South-Western Switzerland (SW-CH)	MK079841		MF765366	this study
6003	2012	*Hymenoscyphus albidus*	Vaud (VD)	46.5	6.383333	South-Western Switzerland (SW-CH)	-		MF765367	this study
6802	2012	*Hymenoscyphus albidus*	Ticino (TI)	45.899583	9.046583	Southern Switzerland (S-CH)	MK079825		MF765368	[35]; this study
6807	2012	*Hymenoscyphus albidus*	Ticino (TI)	45.899583	9.046583	Southern Switzerland (S-CH)	MK079826		MF765372	[35]; this study
6811	2012	*Hymenoscyphus albidus*	Ticino (TI)	45.899583	9.046583	Southern Switzerland (S-CH)	MK079829		MF765374	[35]; this study
6812	2012	*Hymenoscyphus albidus*	Ticino (TI)	45.899583	9.046583	Southern Switzerland (S-CH)	MK079827		MF765375	[35]; this study
6814	2012	*Hymenoscyphus albidus*	Ticino (TI)	45.899583	9.046583	Southern Switzerland (S-CH)	MK079828		MF765377	[35]; this study
8003	2009	*Hymenoscyphus albidus*	Ticino (TI)	46.28244	8.56191	Southern Switzerland (S-CH)	MK079820		-	[35]; this study
8005	2009	*Hymenoscyphus albidus*	Ticino (TI)	46.28244	8.56191	Southern Switzerland (S-CH)	MK079821	MK079851	MF765380	[35]; this study
90723	2009	*Hymenoscyphus albidus*	Glarus (GL)	47.0235	9.04468	Northern Switzerland (N-CH)	MK079815	MK079850	MK079850	this study
90812	2009	*Hymenoscyphus albidus*	Valais (VS)	46.22553	7.37201	South-Western Switzerland (SW-CH)	MK079830		MF765343	this study
CAR10	2012	*Hymenoscyphus albidus*	Britany	47.37256	3.04206	North-Western France (France)	MK079842	MK079847	MF765384	[17]; this study
CAR16	2012	*Hymenoscyphus albidus*	Britany	47.37256	3.04206	North-Western France (France)	MK079843		MF765385	[17]; this study
CAR26	2012	*Hymenoscyphus albidus*	Britany	47.37256	3.04206	North-Western France (France)	MK079844		MF765389	[17]; this study
CAR31	2012	*Hymenoscyphus albidus*	Britany	47.37256	3.04206	North-Western France (France)	MK079845	MK079848	MF765393	[17]; this study
CAR32	2012	*Hymenoscyphus albidus*	Britany	47.37256	3.04206	North-Western France (France)	MK079846		MF765388	[17]; this study
81010	2008	*Hymenoscyphus fraxineus*	Glarus (GL)	47.0235	9.04468	Northern Switzerland (N-CH)	-		MF765432	this study
4330	2009	*Hymenoscyphus fraxineus*	Zurich (ZH)	47.480381	8.37464	Northern Switzerland (N-CH)	-		MF765433	this study
4339	2009	*Hymenoscyphus fraxineus*	Zurich (ZH)	47.480381	8.37464	Northern Switzerland (N-CH)	-		MF765434	this study
4380	2009	*Hymenoscyphus fraxineus*	Zurich (ZH)	47.480381	8.37464	Northern Switzerland (N-CH)	-		MF765435	this study
6006	2012	*Hymenoscyphus fraxineus*	Vaud (VD)	46.5	6.383333	South-Western Switzerland (SW-CH)	-		MF765436	this study

Table A2: List of additional primers used to sequence the full open reading frame of *Hymenoscyphus fraxineus mitovirus 1* and 5′ and 3′ flanking regions of HfMV1. Tm = annealing temperature in degrees Celsius.

Name	Sequence (5'-3')	Tm (⁰C)	Location (bp)	Region
Cf_1F_cc	AAGGCCCCACTCTACTATTA	55	64-666	1
Cf_1R_cc	GTCCACACCTAATTTACCAAG			
Cf_1R2_cc	GGGCAAGAAKWATTTCRTTGT	55	64-646	
Cf_2F_cc	CCGGACTCCCAAAGGTAAT	55	492-1424	2
Cf2R_cc	CGCTTATACTCTGTGGCCAAA			
Cf_2F2_cc	ACGCGCGGACTTAATTACG	55	380-1428	
Cf_2R2_cc	ATACGCTTRTACTCTGTGGC			
Cf_3F_cc	GATTATGACAAAGGAACCGT	58	1244-1749	3
Cf_3R_cc	CATGAAATGAATGCGTAGAA			
Cf_3F2_cc	CAAGAGGGAGTGGAVAAGRT	53	1093-1670	3
Cf_3R2_cc	GCRAGATGAGARAAGAAATGC			
Cf_4F_cc	GTGGGTTTCCTATTGTDGGY	53	1570-2154	4
Cf_4R_cc	GGRCTTGGAGGTAATTTYAA			
Cf_6F2_cc	GCCGCGTTATCAAAAGAAAG	53	2058-2365	6
Cf_6R_cc	CATCGTCCACTGCCACTTAA			

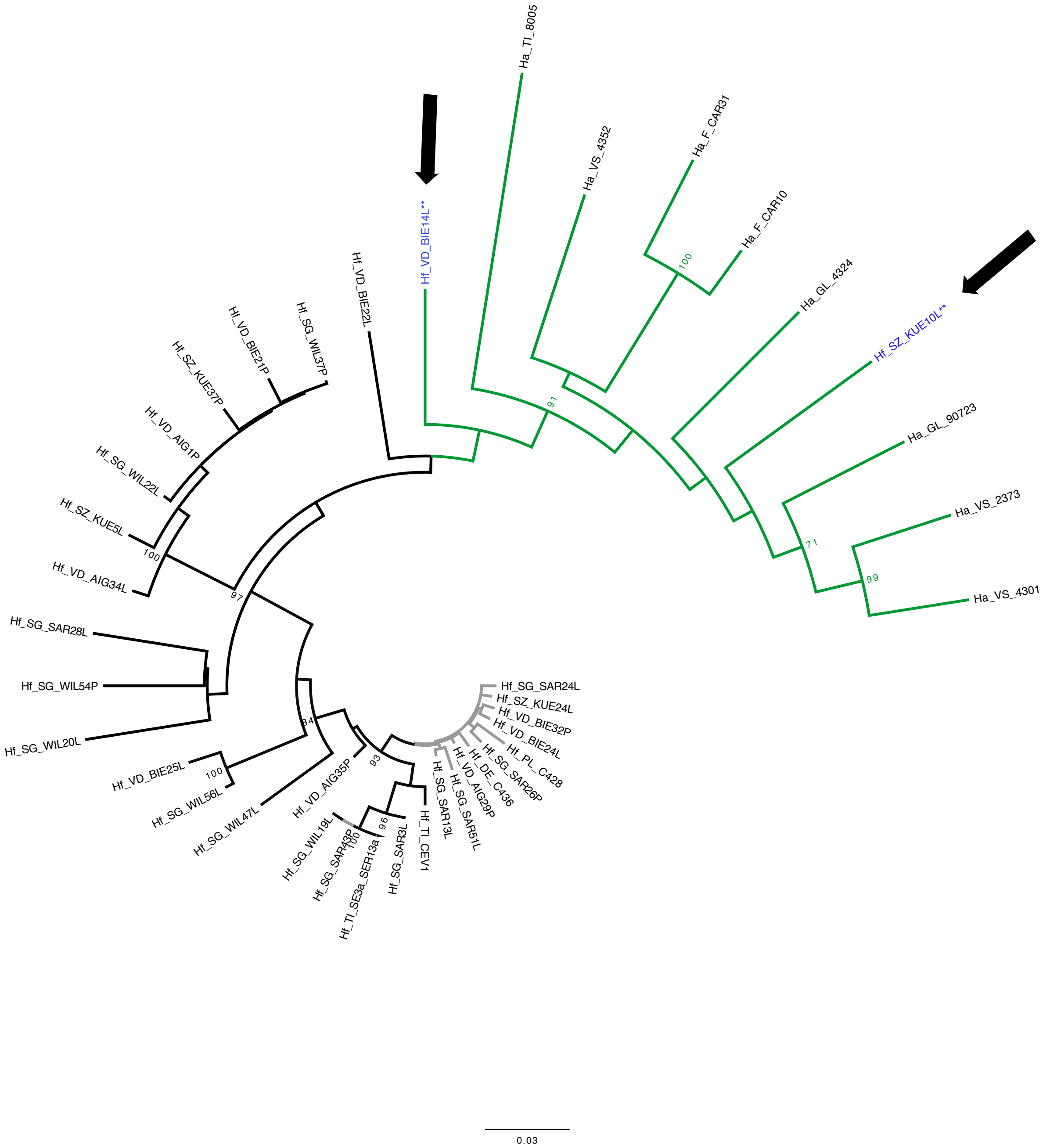

Figure S1: Phylogenetic tree resulting from the RaxML analysis conducted with 40 (8 *H. albidus*, 32 *H. fraxineus*) full sequences (2151 bp) of the RdRP gene of the mitovirus HfMV1. Green color depicts HfMV1 *H. albidus* group isolates. Black color depicts HfMV1 group 2 isolates and grey color HfMV1 group isolates. Blue color and *** mark *H. fraxineus* isolates within the *H. albidus* group. Hf characterizes viral sequences from *H. fraxineus*, Ha from *H. albidus* isolates. For abbreviation see Table 1. Bootstrap values are shown if > 70. Arrows indicate intermixing of *H. albidus* and *H. fraxineus* HfMV1 sequences.

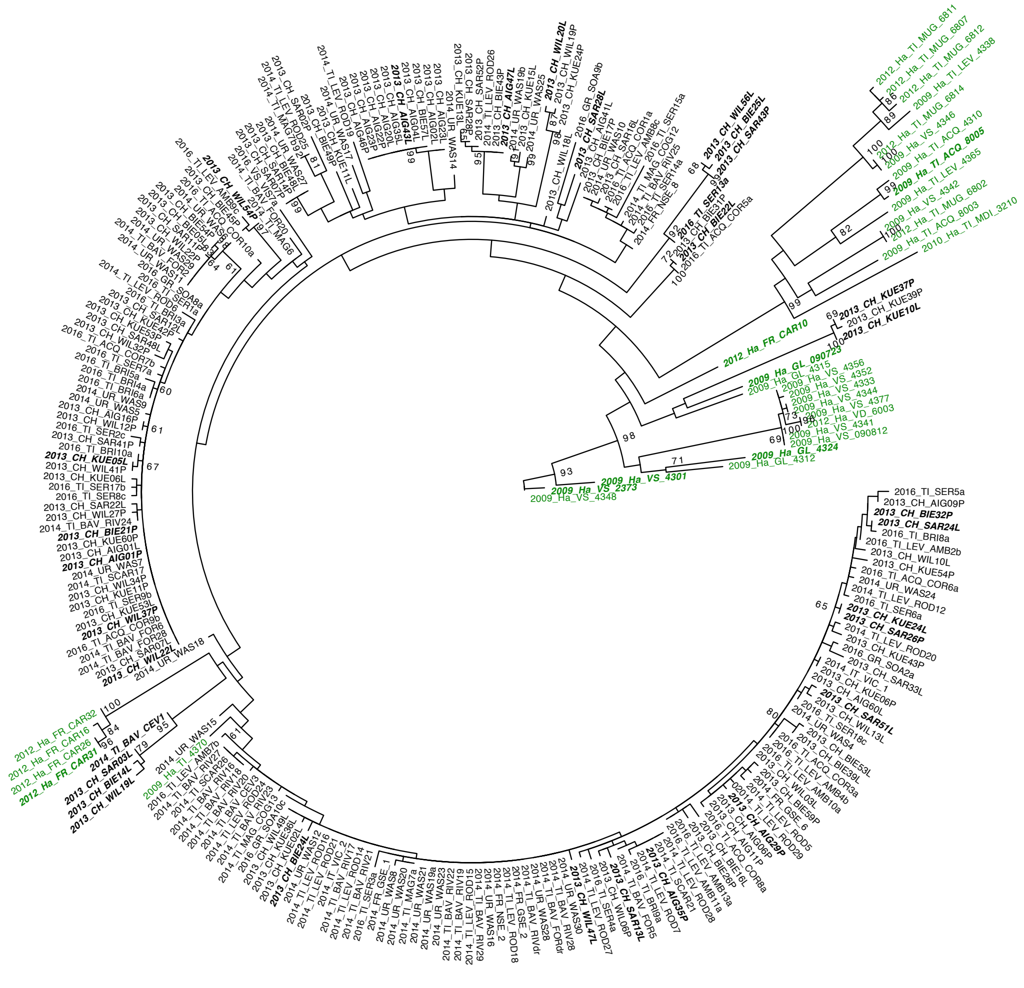

Figure S2: RAxML analysis conducted with 255 sequences (495 bp) of the partial RdRP gene of the mitovirus HfMV1. Green color depicts *H. albidus* isolates. Each sample is named with the sampling year, followed by the sampling locality (for abbreviation see Table 1) and sample name. Bootstrap values are show if ≥ 60. Samples marked in bold and italics are included in the full-length analysis.
